# Immunotherapy plus chemotherapy improves survival in SMARCA4-deficient NSCLC: a real-world cohort study

**DOI:** 10.3389/fimmu.2026.1914598

**Published:** 2026-08-17

**Authors:** YuJun He, YongCun Wang, GuoWei Wu, ShiLiang Li, ZhiBin Lin

**Affiliations:** Department of Pulmonary Oncology, Cancer Hospital, Affiliated Hospital of Guangdong Medical University, Zhanjiang, China

**Keywords:** chemoimmunotherapy, immune checkpoint inhibitors, anti‑angiogenic therapy, real-world study, SMARCA4‑deficient non‑small‑cell lung cancer

## Abstract

**Introduction:**

SMARCA4‑deficient non‑small‑cell lung cancer (SMARCA4‑dNSCLC) is a rare, aggressive NSCLC subtype driven by SWI/SNF complex dysfunction with no standardized therapeutic strategies. Although chemoimmunotherapy represents standard first‑line therapy for driver‑negative advanced NSCLC, its real‑world efficacy in SMARCA4‑dNSCLC remains controversial. SMARCA4 loss frequently generates an immune‑desert tumor microenvironment with low PD‑L1 and sparse CD8⁺ T‑cell infiltration, potentially conferring intrinsic resistance to immune checkpoint inhibitors (ICIs). Pre‑clinical data suggest synergism between anti‑angiogenic agents and chemoimmunotherapy; however, real‑world evidence comparing chemoimmunotherapy alone versus chemoimmunotherapy plus anti‑angiogenics for SMARCA4‑dNSCLC is limited.

**Methods:**

This single‑center retrospective cohort enrolled 22 patients with immunohistochemically‑confirmed advanced SMARCA4‑dNSCLC treated from January 2021 to September 2024. Clinicopathological characteristics, treatment regimens, survival outcomes and safety data were collected. The Kaplan‑Meier method was applied to estimate progression‑free survival (PFS) and overall survival (OS). Given the small sample size, only univariate survival analysis was performed. Objective response rate (ORR) and disease control rate (DCR) were evaluated per RECIST 1.1, and treatment‑related adverse events were graded using CTCAE v4.0. This study obtained local ethics‑committee approval with informed consent waived. The last follow‑up date was September 1, 2025.

**Results:**

Most patients were elderly male smokers with high Ki‑67, frequent adrenal metastasis and low‑to‑negative PD‑L1 expression. Compared with non‑ICI‑based therapy, ICI‑containing regimens achieved significantly superior survival: median OS not reached versus 8.7 months (HR = 0.28, 95%CI:0.08‑0.98, P = 0.047), accompanied by improved PFS, ORR and DCR. Addition of anti‑angiogenic agents brought no extra survival benefit. Elevated CYFRA21‑1 and poor ECOG‑PS predicted unfavorable prognosis. Treatment‑related adverse events were generally tolerable across groups.

**Conclusion:**

Despite an immune‑suppressed tumor microenvironment, real‑world ICI‑containing chemoimmunotherapy delivers meaningful survival benefits for advanced SMARCA4‑dNSCLC, whereas additional anti‑angiogenic combination yields no further clinical advantage. Our findings should be interpreted cautiously given retrospective design and small sample size. Large‑scale multi‑center prospective studies incorporating multivariable prognostic analyses are required to validate these results and optimize individualized treatment for this rare molecular NSCLC subtype.

## 1 Introduction

The mammalian SWItch/Sucrose Non-Fermentable (SWI/SNF) complex serves as a core epigenetic regulator governing tumor initiation and progression via modulation of cell cycle regulation, oncogenic cascades, and metabolic reprogramming (1). As the core catalytic ATPase subunit of the SWI/SNF complex, the *SMARCA4* gene encodes BRG1 protein and mediates ATP hydrolysis to fuel chromatin remodeling-dependent biological functions (2). *SMARCA4* is one of the most frequently mutated genes encoding chromatin-remodeling ATPases across human malignancies. Genetic aberrations of *SMARCA4*, including gene mutation, homozygous deletion and promoter hypermethylation, occur in approximately 5%–7% of all malignant tumors (3). Approximately 5%–10% of all non-small cell lung cancer (NSCLC) cases harbor loss-of-function *SMARCA4* deficiency, and SMARCA4-dNSCLC is characterized by highly aggressive biological phenotypes (4, 5). Rigorous biological distinction is required between SMARCA4-deficient NSCLC (defined by IHC-confirmed BRG1 nuclear loss) and sequencing-confirmed SMARCA4-mutant NSCLC. These entities are diagnostically distinct and non-overlapping: some mutants retain BRG1 expression, while rare deficient cases lack detectable genomic alterations. Clinically, SMARCA4 deficiency correlates with a more aggressive phenotype, characterized by an “immune-desert” microenvironment and heightened proliferation, distinguishing it from mere mutation. *SMARCA4* deficiency can accelerate tumor progression, increase the incidence and burden of metastasis, and lead to poor prognosis in patients (4, 6). Over 75% of SMARCA4-dNSCLC patients are diagnosed at stage III–IV upon initial presentation (6, 7).

Beyond these innate adverse biological features, this subtype also displays heterogeneous therapeutic responsiveness relative to SMARCA4 wild-type NSCLC, rather than universal treatment resistance. While SMARCA4-deficient tumors show substantially attenuated anti-tumor activity when treated with single-agent immunotherapy, combination regimens incorporating chemotherapy can partially reverse this therapeutic disadvantage (8). Consistently, published cohort data report markedly shortened median OS in patients with SMARCA4 protein loss versus wild-type counterparts (15.6 months vs 25.6 months, P < 0.01), a survival gap shaped jointly by the tumor’s native invasive biology and inferior response to single-modality therapy (9).

Currently, no standardized therapeutic guidelines exist for SMARCA4-dNSCLC. Surgery, radiotherapy and chemotherapy remain conventional therapeutic modalities for SMARCA4-dNSCLC. *SMARCA4* alteration also serves as an adverse prognostic risk factor after curative resection in operable NSCLC patients (10). In a previous cohort consisting of 42 stage I–II resected NSCLC patients, significantly shorter recurrence-free survival (RFS) was observed in patients harboring *SMARCA4* alteration compared with wild-type counterparts (4.5 months vs 28.5 months, *P* = 0.0007) (11). The lack of actionable driver alterations, coupled with the intrinsically undruggable nature of the *SMARCA4* gene, renders molecular targeted therapy largely ineffective (8, 12). Although preclinical studies have identified specific vulnerabilities, these agents remain confined to investigational settings without routine clinical adoption. Consequently, platinum-based chemotherapy constitutes the mainstay of systemic treatment (13); however, this subtype often exhibits inherent chemoresistance, resulting in suboptimal outcomes with single-agent regimens.

Based on landmark phase III trials including IMpower110, KEYNOTE-189 and KEYNOTE-407, immune checkpoint inhibitor (ICI)-centered chemoimmunotherapy has evolved into standard first-line therapy for advanced driver-negative NSCLC (14–16). However, the clinical efficacy of ICIs in SMARCA4-dNSCLC remains controversial. Accumulating evidence indicates that SMARCA4-dNSCLC often presents an immune-desert tumor microenvironment (TME) (17), accompanied by low PD-L1 expression (5, 18), diminished tumor mutational burden (TMB), and sparse intratumoral immune cell infiltration, all of which potentially blunt single-agent ICI efficacy. *SMARCA4* deficiency results in reduced CD8^+^ T cell infiltration (19). Notably, in tumors with concurrent *KRAS* mutations, multiplex immunofluorescence demonstrates diminished numbers of both PD-1^+^ immune cells and PD-1^+^CD8^+^ double-positive T cells (9). While emerging studies have preliminarily uncovered the unique immunological landscape of this rare subtype, optimal therapeutic strategies have not yet been well-established. Although these features suggest intrinsic resistance to single-agent ICI, several retrospective studies have reported promising survival benefits from ICI-based regimens in this subtype (20–22). Compared with other treatments, patients with *BRG1*-deficient NSCLC demonstrate a significantly longer progression-free survival (PFS) of 8.2 months following first-line ICI combination chemotherapy, compared with 3.5 months with chemotherapy alone (23). Moreover, immunotherapy is associated with improved overall survival (OS) (5, 23). These studies underscore the intricate mechanisms governing immunotherapy responsiveness in this subtype. Preclinical findings have validated that anti-angiogenic drugs normalize aberrant tumor vasculature, remodel the immunosuppressive TME, and synergize with chemoimmunotherapy to potentiate anti-tumor activity (24, 25). Nonetheless, real-world data comparing chemoimmunotherapy alone versus chemoimmunotherapy combined with anti-angiogenic agents in SMARCA4-dNSCLC remain extremely scarce.

Given the rarity of SMARCA4-dNSCLC and the lack of consistent real-world evidence regarding ICI efficacy and combined treatment strategies, we conducted a retrospective cohort study of 22 patients with advanced SMARCA4-dNSCLC. We comprehensively compared clinicopathological features, treatment responses, survival outcomes, and safety profiles between ICI-based regimens and conventional non-ICI therapies. Additionally, we explored potential prognostic factors and evaluated the incremental value of adding anti-angiogenic therapy to chemoimmunotherapy. This study aims to provide supplementary real-world clinical data to support individualized management for this rare and aggressive NSCLC subtype.

## 2 Materials and methods

### 2.1 Study population and data collection

This was a single-center retrospective cohort study. A total of 22 patients with SMARCA4-dNSCLC were retrospectively enrolled from the Scientific Research Big Data Platform Center of the Affiliated Hospital of Guangdong Medical University. Eligible patients received treatment between January 2021 and September 2024, with a confirmed diagnosis of SMARCA4/BRG1 deficiency via immunohistochemistry (IHC) and complete clinical treatment and follow-up data. SMARCA4/BRG1 deficiency was defined as the complete loss or significant reduction of nuclear staining in tumor cells based on IHC detection. Tumor staging was performed in accordance with the 8th edition of the American Joint Committee on Cancer (AJCC) TNM staging system for NSCLC. Follow-up information was collected through inpatient medical record review and telephone interviews, with the last follow-up date set as September 1, 2025.

All first-line systemic treatment regimens were prospectively formulated by thoracic oncologists or thoracic multidisciplinary tumor board (MDT), in strict accordance with domestic and international clinical guidelines for non-small cell lung cancer (NSCLC) issued between 2021 and 2024. Regimen selection comprehensively incorporated multiple clinical and molecular indicators including tumor stage, molecular biomarkers, performance status, tumor burden and underlying comorbidities, with hierarchical treatment criteria specified as follows: Early and intermediate-stage patients: Comprehensive radical therapy centered on surgical resection was administered, with neoadjuvant or postoperative adjuvant therapy matched according to tumor stage and high-risk recurrence factors. Advanced patients with actionable driver alterations: Individuals harboring targetable mutations such as EGFR received corresponding single-agent small-molecule targeted therapy as priority. Advanced driver-negative patients: Platinum-based chemoimmunotherapy was recommended as standard first-line therapy per guidelines, with regimens stratified by PD-L1 expression status. Patients with tumor proportion score (TPS) ≥50% received single-agent immune checkpoint inhibitor (ICI); those with low or negative PD-L1 expression were treated with platinum plus ICI; patients without evaluable PD-L1 testing were assigned chemoimmunotherapy or single platinum chemotherapy. ECOG performance status stratification: Patients with ECOG 0–1 and favorable physical tolerance received full-dose ICI combination regimens, whereas those with ECOG ≥2 and poor tolerance were administered single-agent chemotherapy or single-agent ICI only. Tumor burden stratification: Chemoimmunotherapy plus anti-angiogenic therapy was prioritized for advanced patients with extensive visceral metastases, high tumor burden or rapidly progressive symptomatic disease to achieve rapid tumor control. Additionally, ICI was contraindicated for patients complicated with severe organ dysfunction, active uncontrolled infection or hematological disorders, who received chemotherapy alone; complex cases with ambiguous biomarkers or multiple comorbidities were reviewed by MDT, and all therapeutic decisions fully complied with standardized institutional protocols with minimal discretionary physician adjustment.

### 2.2 Data collection

Collected clinical variables included patient demographics (age, sex), smoking history, Eastern Cooperative Oncology Group (ECOG) performance status (PS) at initial diagnosis, tumor histological type, AJCC 8th-edition TNM stage, metastatic sites, treatment modalities (surgery, radiotherapy, chemotherapy, immunotherapy, and targeted therapy), and prior treatment lines before immunotherapy initiation. Pathological and immunohistochemical markers (Ki-67 index, PD-L1 expression) and serum tumor markers (CEA, CYFRA21-1, NSE, SCC) were systematically recorded.

For patients receiving immunotherapy, detailed clinical information was collected, including the specific immune checkpoint inhibitor (ICI) administered, treatment regimens, number of treatment cycles, and treatment-related adverse events (TRAEs). All TRAEs were graded according to the National Cancer Institute Common Terminology Criteria for Adverse Events (CTCAE) version 4.0.

Treatment efficacy was evaluated based on the objective response rate (ORR) and disease control rate (DCR), where DCR was defined as the sum of complete response (CR), partial response (PR), and stable disease (SD), in accordance with the Response Evaluation Criteria in Solid Tumors (RECIST) version 1.1. The primary study endpoints were progression-free survival (PFS) and overall survival (OS). PFS was defined as the interval from treatment initiation to disease progression or all-cause death, whichever occurred first. OS was defined as the time from treatment initiation to all-cause death.

This study was approved by the Ethics Committee of the Affiliated Hospital of Guangdong Medical University and conducted in strict accordance with the principles of the Declaration of Helsinki. Informed patient consent was waived due to the retrospective nature of the study design.

### 2.3 Statistical analysis

Categorical variables were presented as frequencies and percentages, while continuous variables were described using median, minimum, and maximum values. Kaplan–Meier survival analysis was performed to estimate PFS and OS, with median survival outcomes and corresponding two-sided 95% confidence intervals (CIs) calculated for all groups. Survival rates at different time points were also summarized with two-sided 95% CIs.

Given the relatively small sample size of the present study, multivariate regression analysis was not feasible, and only univariate survival analyses were conducted. All results should be interpreted cautiously with consideration of potential confounding biases inherent to retrospective observational studies.

## 3 Results

### 3.1 Patient characteristics

A total of 22 patients with SMARCA4-dNSCLC were included in this study, with 15 patients in the ICI group and 7 patients in the non-ICI group. The baseline demographic, clinical, pathological, and treatment-related characteristics are summarized in **Table 1**.

**Table 1:** Baseline characteristics and contrast of clinical factors in patients with SMARCA4-deficient NSCLC between the ICI and non-ICI cohorts.

Factors	Total (n=22)	Non-ICI (n=7)	ICI (n=15)	*P*
Gender, n(%)				0.96
Female	1 (4.55)	1 (14.29)	0 (0.00)	
Male	21 (95.45)	6 (85.71)	15 (100.00)	
Median age at				1.00
diagnosis (years)				
<60	7(31.82)	2 (28.57)	5(33.33)	
≥60	15(68.18)	5(71.43)	10(66.67)	
Smoking history, n(%)				1.00
No	9(40.91)	3(42.86)	6(40.00)	
Yes	13(59.09)	4(57.14)	9(60.00)	
Performance status, n(%)				0.565
0–1	18 (81.82)	5 (71.43)	13 (86.67)	
>1	4 (18.18)	2 (28.57)	2 (13.33)	
Clinical stage				0.062
I	2 (9.09)	2 (28.57)	0 (0.00)	
II	1 (4.55)	0 (0.00)	1 (6.67)	
III	4 (18.18)	2 (28.57)	2 (13.33)	
IV	15 (68.18)	3 (42.86)	12 (80.00)	
T				0.597
1	4 (18.18)	2 (28.57)	2 (13.33)	
2	5 (22.73)	1 (14.29)	4 (26.67)	
3	3 (13.64)	0 (0.00)	3 (20.00)	
4	10 (45.45)	4 (57.14)	6 (40.00	
N, n(%)				0.127
0	5 (22.73)	3 (42.86)	2 (13.33)	
1	1 (4.55)	0 (0.00)	1 (6.67)	
2	6 (27.27)	3 (42.86)	3 (20.00)	
3	10 (45.45)	1 (14.29)	9 (60.00)	
M, n(%)				0.145
0	7 (31.82)	4 (57.14)	3 (20.00)	
1	15 (68.18)	3 (42.86)	12 (80.00)	
Pleural Effusion				1.00
No	19 (86.36)	6 (85.71)	13 (86.67)	
Yes	3 (13.64)	1 (14.29)	2 (13.33)	
Intralung metastasis				
No	12 (54.55)	4 (57.14)	8 (53.33)	1.00
Yes	10 (45.45)	3 (42.86)	7 (46.67)	
Bone metastases				1.00
No	14 (63.64)	5 (71.43)	9 (60.00)	
Yes	8 (36.36)	2 (28.57)	6 (40.00)	
Brain metastases				
No	17 (77.27)	6 (85.71)	11 (73.33)	1.00
Yes	5 (22.73)	1 (14.29)	4 (26.67)	
Adrenal metastases				
No	19 (86.36)	7 (100.00)	12 (80.00)	0.523
Yes	3 (13.64)	0 (0.00)	3 (20.00)	
Pleural metastasis				
No	17 (77.27)	7 (100.00)	10 (66.67)	0.135
Yes	5 (22.73)	0 (0.00)	5 (33.33)	
Histological type				0.027
NSCLC, not otherwise specified	8 (36.36)	0 (0.00)	8 (53.33)	
Adenocarcinoma	13 (59.09)	7 (100.00)	6 (40.00)	
Sarcomatoid carcinoma	1 (4.55)	0 (0.00)	1 (6.67)	
PD-L1	11(50%)	4(36.36)	7(63.64)	1.00
<1%	7 (63.64)	3 (75.00)	4 (57.14)	
1%~49%	4 (36.36)	1 (25.00)	3 (42.86)	
>49%	0 (0.00)	0 (0.00)	0 (0.00)	
Ki-67				0.274
≤30%	5 (22.73)	3 (42.86)	2 (13.33)	
>30%	17 (77.27)	4 (57.14)	13 (86.67)	
EGFR mutation, n (%)				
	22 (77.27)	5 (71.43)	15 (100)	
	2 (9.09)	2 (28.57)	0 (0.00	
NSE				1.00
Normal	13 (59.09)	4 (57.14)	9 (60.00	
Elevated	9 (40.91)	3 (42.86)	6 (40.00)	
CEA				0.652
Normal	12 (54.55)	3 (42.86)	9 (60.00)	
Elevated	10 (45.45)	4 (57.14)	6 (40.00)	
CYFRA21-1				1.00
Normal	5 (22.73)	1 (14.29)	4 (26.67)	
Elevated	17 (77.27)	6 (85.71)	11 (73.33)	
SCC				1.00
Normal	20 (90.91)	7(100.00)	13 (86.67)	
Elevated	2 (9.09)	0 (0.00)	2 (13.33)	
Surgery				0.334
No	16 (72.73)	4 (57.14)	12 (80.00)	
Yes	6 (27.27)	3 (42.86)	3 (20.00)	
Chest Radiotherapy				0.630
No	15 (68.18)	4 (57.14)	11 (73.33)	
Yes	7 (31.82)	3 (42.86)	4 (26.67)	
Chemotherapy				0.077
No	4 (18.18)	3 (42.86)	1 (6.67)	
Yes	18 (81.82)	4 (57.14)	14 (93.33)	
Anti-angiogenic targeted therapy				0.630
No	15 (68.18)	4 (57.14)	11 (73.33)	
Yes	7 (31.82)	3 (42.86)	4 (26.67)	

The cohort was predominantly male (21/22, 95.45%), with a median age at diagnosis ≥ 60 years in 15 patients (68.18%). Thirteen patients (59.09%) had a history of smoking. Most patients had an Eastern Cooperative Oncology Group performance status of 0–1 (18/22, 81.82%) and stage IV disease (15/22, 68.18%). The histological subtype distribution differed significantly between the ICI and non-ICI groups (P = 0.027). Among the total 22 enrolled SMARCA4-dNSCLC patients, adenocarcinoma accounted for the largest proportion (13/22, 59.09%), followed by NSCLC not otherwise specified (NOS, 8/22, 36.36%), and sarcomatoid carcinoma only occupied a minor fraction (1/22, 4.55%). Notably, all seven patients in the non-ICI cohort were pathologically confirmed as adenocarcinoma (7/7, 100.00%). In contrast, the ICI group (n=15) consisted of 6 adenocarcinoma cases (40.00%), 8 NSCLC-NOS cases (53.33%), and 1 sarcomatoid carcinoma case (6.67%). The uneven histological composition between the two treatment arms constituted the only statistically significant intergroup discrepancy in baseline clinical features.

Regarding molecular biomarkers, only 11 patients completed PD-L1 testing. Seven patients (63.64%) were PD-L1 negative (TPS < 1%), and four patients (36.36%) had low PD-L1 expression (TPS 1%–49%), with no cases showing expression ≥ 50%. The Ki-67 proliferation index was > 30% in 17 patients (77.27%). Serum tumor markers were frequently elevated, including CYFRA21–1 in 17 patients (77.27%), CEA in 10 patients (45.45%), NSE in 9 patients (40.91%), and SCC in 2 patients (9.09%). No significant between-group differences were observed in PD-L1 expression, Ki-67 index, or tumor marker levels. Genomic profiling of common targetable driver alterations, including EGFR, ALK, ROS1, KRAS and MET, was conducted across all enrolled patients. EGFR mutations were identified in 2 patients (9.1%), whereas no other canonical driver molecular aberrations were detected in the remaining 20 cases (90.9%). These two EGFR-mutant patients were excluded from subsequent survival analyses.

In terms of prior treatment history, 6 patients (27.27%) had undergone surgery, 7 (31.82%) had received chest radiotherapy, 18 (81.82%) had received chemotherapy, and 7 (31.82%) had received anti-angiogenic targeted therapy. There were no significant differences in prior treatment modalities between the ICI and non-ICI groups.

Other clinical characteristics, including tumor stage, T/N/M classification, pleural effusion, and metastatic patterns (intralung, bone, brain, and adrenal metastases), were balanced between the two groups. Except for histological subtype, all baseline characteristics were well-balanced between the ICI and non-ICI groups (all *P* > 0.05).

### 3.2 Efficacy response

This study included 22 patients with SMARCA4-dNSCLC who received systemic therapy. Among these patients, two achieved a complete response (CR) and six achieved a partial response (PR), yielding an overall objective response rate (ORR) of 34.7% (8/22) and a disease control rate (DCR) of 50.0% (11/22).

Subgroup analyses demonstrated that ORR and DCR were comparable across most clinical and pathological variables. The only significant intergroup difference was identified for serum CYFRA21–1 status. Patients with elevated CYFRA21–1 levels exhibited significantly lower ORR (23.5% vs. 80.0%, *P* = 0.039) and DCR (35.3% vs. 100.0%, *P* = 0.035) relative to patients with normal CYFRA21–1 levels. No significant differences in ORR or DCR were observed with respect to age, sex, smoking history, ECOG performance status, clinical stage, T/N/M classification, pleural effusion, intrapulmonary metastasis, bone metastasis, brain metastasis, adrenal metastasis, pleural metastasis, histological subtype, PD-L1 expression, Ki-67 index, NSE, CEA, SCC, and prior treatment histories including surgery, radiotherapy, chemotherapy, anti-angiogenic therapy, and different combination regimens (all *P* > 0.05).

Stratified analysis by treatment modality revealed that patients receiving ICI-based regimens had numerically higher ORR (46.7% vs. 14.3%) and DCR (60.0% vs. 28.6%) than those treated with non-ICI regimens, though the differences were not statistically significant (*P* = 0.193 for both ORR and DCR). Similarly, patients treated with chemoimmunotherapy showed a marginal trend toward superior ORR (60.0% vs. 16.7%) and DCR (70.0% vs. 33.3%) compared with patients receiving non-immunotherapy regimens, without statistical significance (*P* = 0.074 for ORR, *P* = 0.198 for DCR). In addition, treatment responses to chemotherapy combined with anti-angiogenic therapy and chemoimmunotherapy combined with anti-angiogenic therapy were comparable across groups (all *P* > 0.05). The assessments of the efficacy evaluation for each clinical characteristic subgroup are presented in **Table 2**.

**Table 2:** Evaluation of ORR and DCR across several clinical features and treatment method categories.

Features	n	Objective response rate			Disease control rate		
		n	Rate (%)	*P*	n	Rate (%)	*P*
Overall Rate	22	8	34.7		11		
Gender, n(%)				1.00			1.00
Female	21	8	38.09		11	52.38	
Male	1	0	0.00		0	0	
Median age at							
diagnosis (years)				1.00			0.361
<60	7	2	28.57		5	71.43	
≥60	15	6	40.00		6	40.00	
Smoking history, n(%)				0.380			0.387
No	9	2	22.22		3	33.33	
Yes	13	6	46.15		8	61.54	
Performance status, n(%)				0.254			0.09
0–1	18	8	44.44		11	61.11	
>1	4	0	0.00		0	0.00	
Clinical stage				0.394			0.635
I	2	1	50.00		1	50.00	
II	1	1	100.00		1	100.00	
III	4	2	50.00		3	75.00	
IV	15	4	26.67		6	40.00	
T				0.389			0.482
1	4	3	75.00		3	75.00	
2	5	1	20.00		1	20.00	
3	3	1	33.33		2	66.67	
4	10	3	30.00		5	50.00	
N, n(%)				0.540			0.631
0	5	2	40.00		3	60.00	
1	1	0	0.00		0	0.00	
2	6	1	16.67		2	33.33	
3	10	5	50.00		6	60.00	
M, n(%)				0.343			0.361
0	7	4	57.14		5	71.43	
1	15	4	26.67		6	40.00	
Pleural Effusion				1.00			0.635
No	16	6	37.50		9	56.25	
Yes	6	2	33.33		2	33.33	
Intralung Metastasis				0.204			0.67
No	12	6	50.00		7	58.33	
Yes	10	2	20.00		4	40.00	
Bone metastases				0.386			1.00
No	14	4	28.57		7	50.00	
Yes	8	4	50.00		4	50.00	
Brain metastases				0.613			1.00
No	17	7	41.18		8	47.06	
Yes	5	1	20.00		3	60.00	
Adrenal metastases				1.00			1.00
No	19	7	36.84		9	47.37	
Yes	3	1	33.33		2	66.67	
Pleural metastasis				1.00			1.00
No	17	6	35.29		8	47.06	
Yes	5	2	40.00		3	60.00	
Histological type				0.775			0.183
NSCLC, not otherwise specified	8	4	50.00		6	54.55	
Adenocarcinoma	13	4	30.76		5	38.46	
Sarcomatoid carcinoma	1	0	0.00		0	0.00	
PD-L1	11			0.236	11		0.236
<1%	7	3	42.86		3	42.86	
1%~49%	4	0	0.00		0	0.00	
Ki-67				0.613			1.00
≤30%	5	1	20.00		2	40.00	
>30%	17	7	41.18		9	52.94	
NSE				0.662			1.00
Normal	13	4	30.77		6	46.15	
Elevated	9	4	44.44		5	55.56	
CEA				0.675			0.67
Normal	12	5	41.67		7	58.33	
Elevated	10	3	30.00		4	40.00	
CYFRA21-1				0.039			0.035
Normal	5	4	80.00		5	100.00	
Elevated	17	4	23.53		6	35.29	
SCC				0.515			0.476
Normal	20	8	40.00		11	55.00	
Elevated	2	0	0.00		0	0.00	
Chest Surgery				0.624			1.00
No	16	5	31.25		8	50.00	
Yes	6	3	50.00		3	50.00	
Immunotherapy				0.193			0.361
No	7	1	14.28		2	28.57	
Yes	15	7	46.67		9	60.00	
Chest Radiotherapy				0.193			1.00
No	15	7	46.67		7	46.67	
Yes	7	1	14.29		4	57.14	
Chemotherapy				1.00			0.586
No	4	1	25.00		1	25.00	
Yes	18	7	38.89		11	61.11	
Anti-angiogenic targeted therapy				0.351			1.00
No	15	7	46.67		8	53.33	
Yes	7	1	14.29		3	42.86	
Chemotherapy plus immunotherapy				0.074			0.198
No	12	2	16.67		4	33.33	
Yes	10	6	60.00		7	70.00	
Chemotherapy plus anti-angiogenic therapy				0.273			1.00
No	19	8	42.11		10	52.63	
Yes	3	0	0.00		1	33.33	
Chemotherapy plus immunotherapy and anti-angiogenic therapy				1.00			
No	18	7	38.89		9	50.00	
Yes	4	1	25.00		2	50.00	

### 3.3 Survival analysis

The last follow-up date was September 1, 2025, with a median follow-up duration of 18.40 months (range: 2.14–51.38 months). For the 22 enrolled patients, the median progression-free survival (mPFS) and median overall survival (mOS) were 27.9 months and 31.4 months, respectively. During the follow-up period, 11 patients (50%) experienced disease progression, and 10 patients (45.45%) died of the disease. No treatment-related deaths occurred in this cohort.

Kaplan–Meier survival analysis showed that patients with SMARCA4-dNSCLC aged >60 years had numerically inferior PFS and OS compared with patients aged ≤60 years. The median PFS was not reached in younger patients versus 11.7 months in elderly patients (HR = 2.73, 95% CI: 0.59–12.71, *P* = 0.200). Similarly, the median OS was unreached in the ≤60 years group versus 15.7 months in the >60 years group (HR = 5.43, 95% CI: 0.68–43.67, *P* = 0.111), with no significant intergroup differences.

**Figure 1:**
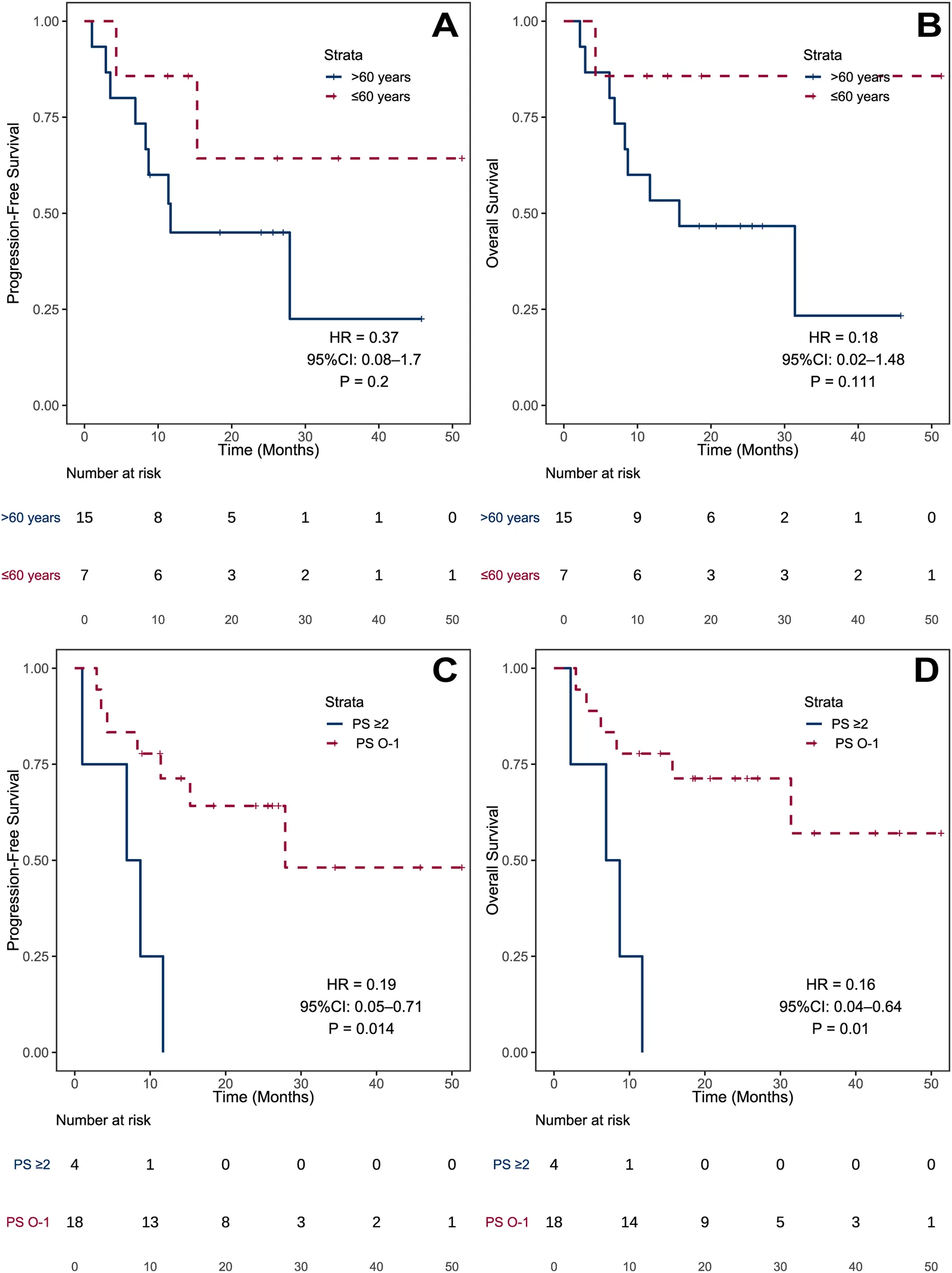
Kaplan–Meier curves of progression-free survival (PFS) and overall survival (OS) stratified by age and Eastern Cooperative Oncology Group (ECOG) performance status (PS) in patients with *SMARCA4*-deficient NSCLC. Panels **(A, B)** show PFS and OS stratified by age (≤60 years, blue line; >60 years, red line); corresponding number-at-risk tables are shown below each curve. Median PFS was not reached (NR) in patients ≤60 years vs 11.7 months for those >60 years (HR = 2.73, 95% CI: 0.59–12.71, *P* = 0.200). Median OS was NR vs 15.7 months (HR = 5.43, 95% CI: 0.68–43.67, *P* = 0.111). Panels **(C, D)** present PFS and OS stratified by ECOG PS (0–1, blue line; ≥2, red line) with number-at-risk tables. Median PFS was 27.9 months (PS 0–1) vs 7.8 months (PS ≥2; HR = 5.34, 95% CI: 1.41–20.22, *P* = 0.014). Median OS was NR (PS 0–1) vs 7.8 months (PS ≥2; HR = 6.37, 95% CI: 1.57–25.89, *P* = 0.010).

In terms of Eastern Cooperative Oncology Group (ECOG) performance status, patients with PS ≥ 2 exhibited significantly poorer survival outcomes than those with PS 0–1. The median PFS was not attained in the PS 0–1 cohort versus 7.8 months in the PS ≥ 2 cohort (HR = 5.34, 95% CI: 1.41–20.22, *P* = 0.014). The median OS was also unreached in the PS 0–1 group versus 7.8 months in the PS ≥ 2 group (HR = 6.37, 95% CI: 1.41–20.22, *P* = 0.010). The corresponding Kaplan–Meier curves and risk tables are presented in **Figure 1**.

**Figure 2:**
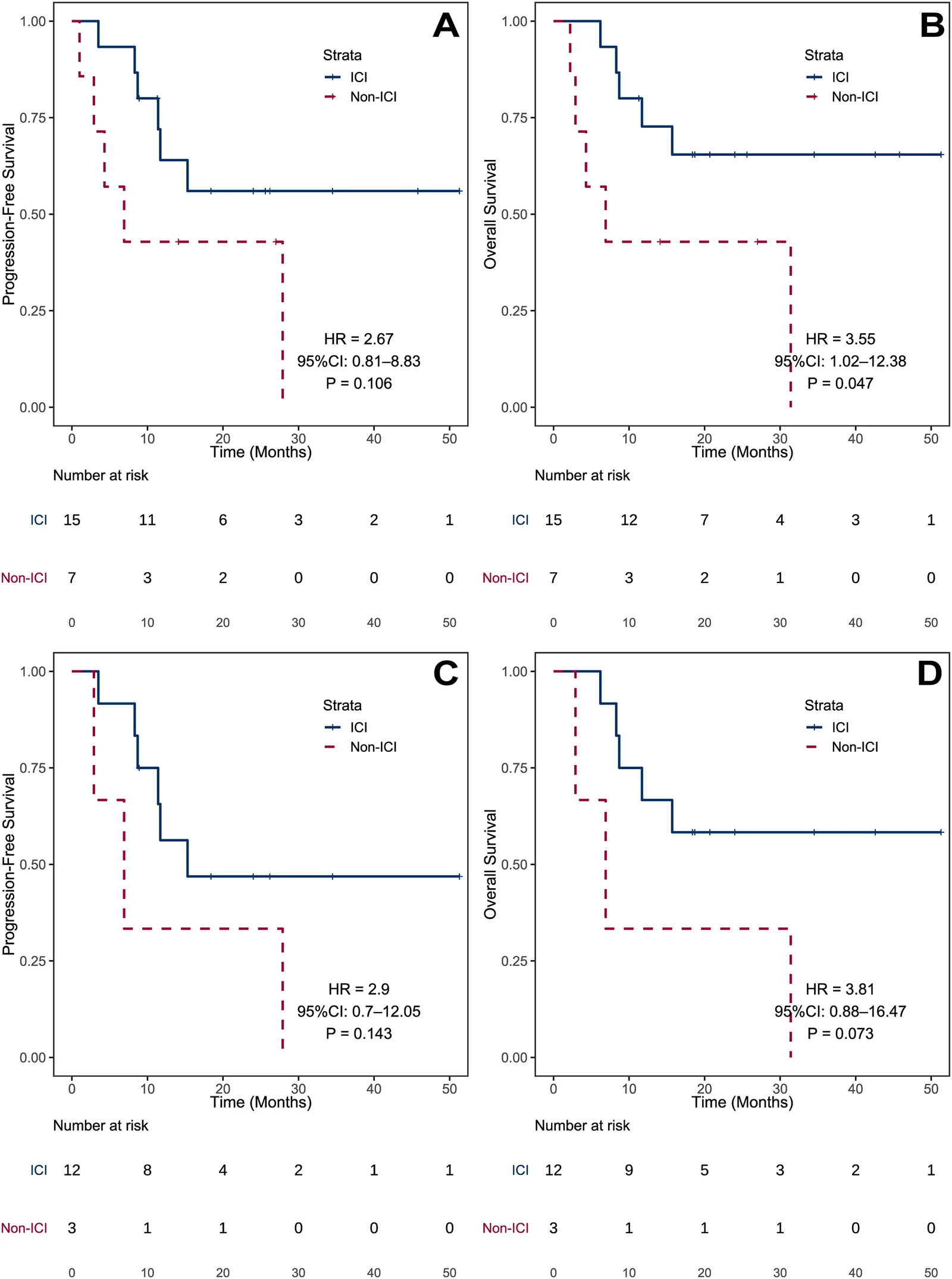
Kaplan–Meier PFS and OS curves stratified by immune checkpoint inhibitor (ICI) therapy in patients with *SMARCA4*-deficient NSCLC. Panels **(A, B)** show full cohort comparisons: Non-ICI (blue) vs ICI (red), with risk tables below each plot. Median PFS was 6.9 months (Non-ICI) vs not reached (NR, ICI; HR = 0.37, 95% CI: 0.11–1.23, *P* = 0.106); median OS was 6.9 months vs NR (HR = 0.28, 95% CI: 0.08–0.98, *P* = 0.047). Panels **(C, D)** restrict analysis to stage IV patients. Median PFS was 6.9 months (Non-ICI) vs 15.3 months (ICI; HR = 0.34, 95% CI: 0.08–1.43, *P* = 0.143); median OS was 6.9 months vs NR (HR = 0.26, 95% CI: 0.06–1.13, *P* = 0.073). Number-at-risk tables are displayed under all curves.

Survival outcomes were further compared between patients receiving ICI-based therapy and non-ICI therapy (**Figure 2**). In the overall cohort, ICI treatment conferred numerically prolonged PFS compared with non-ICI treatment, though the difference was not statistically significant (median PFS: NR vs. 8.7 months, HR = 0.37, 95% CI: 0.11–1.23, *P* = 0.106). Notably, patients in the ICI group achieved significantly improved OS, with an unreached median OS compared with 8.7 months in the non-ICI group (HR = 0.28, 95% CI: 0.08–0.98, *P* = 0.047). Subgroup analysis restricted to patients with stage IV disease yielded consistent survival trends. Compared with non-ICI therapy, ICI-containing treatment was associated with numerically superior PFS and OS, without reaching statistical significance (PFS: HR = 0.34, 95% CI: 0.08–1.43, *P* = 0.143; OS: HR = 0.26, 95% CI: 0.06–1.13, *P* = 0.073).

**Figure 3:**
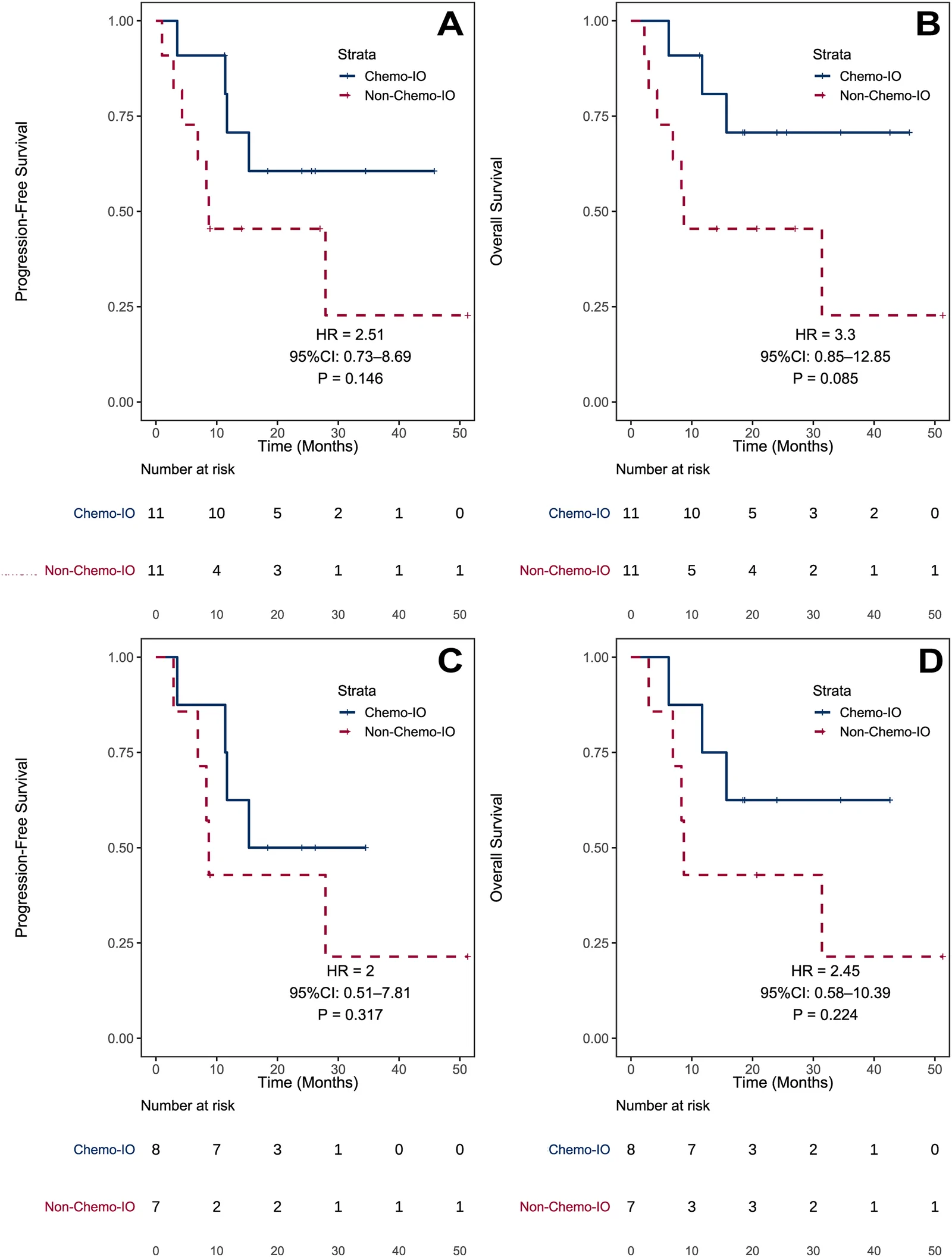
Kaplan–Meier PFS and OS curves stratified by chemoimmunotherapy (Chemo-IO) in patients with *SMARCA4*-deficient NSCLC. Panels **(A, B)** show full cohort comparisons: Non-Chemo-IO (blue) vs Chemo-IO (red), with risk tables below each plot. Median PFS was 8.7 months (Non-Chemo-IO) vs not reached (NR, Chemo-IO; HR = 0.40, 95% CI: 0.12–1.38, *P* = 0.146); median OS was 8.7 months vs NR (HR = 0.30, 95% CI: 0.08–1.18, *P* = 0.085). Panels **(C, D)** restrict analysis to stage IV patients. Median PFS was 8.7 months (Non-Chemo-IO) vs NR (Chemo-IO; HR = 0.50, 95% CI: 0.13–1.95, *P* = 0.317); median OS was 8.7 months vs NR (HR = 0.41, 95% CI: 0.10–1.73, *P* = 0.224). Number-at-risk tables are displayed under all curves.

Additionally, we conducted prognostic analyses of PFS and OS for patients categorized by receiving chemoimmunotherapy (Chemo-IO) and non-chemoimmunotherapy (Non-Chemo-IO) (**Figure 3**). In the overall cohort, patients treated with Chemo-IO achieved numerically longer PFS and OS versus Non-Chemo-IO counterparts, yet no statistically significant differences were detected. Median PFS was not reached for Chemo-IO versus 8.7 months for Non-Chemo-IO (HR = 0.40, 95% CI: 0.12–1.38, *P* = 0.146), while median OS was also unreached in Chemo-IO group versus 8.7 months in Non-Chemo-IO group (HR = 0.30, 95% CI: 0.08–1.18, *P* = 0.085). Subgroup analysis limited to stage IV patients displayed consistent survival trends. Numerically superior PFS and OS were observed in Chemo-IO arm without statistical significance (PFS: HR = 0.50, 95% CI: 0.13–1.95, P = 0.317; OS: HR = 0.41, 95% CI: 0.10–1.73, *P* = 0.224). Of note, the wide confidence interval of the hazard ratio reflects the limited sample size of this cohort. This exploratory finding should be regarded as hypothesis-generating rather than a definitive estimate of treatment effect.

Further exploratory subgroup survival analysis was performed to evaluate whether additional anti-angiogenic therapy could improve survival on the basis of chemo-IO regimen in stage IV SMARCA4-dNSCLC patients (**Supplementary Figure 1**). No obvious difference in PFS or OS was observed between patients receiving chemo-IO monotherapy and chemo-IO plus anti-angiogenic combination. Median PFS and OS were not reached in both cohorts, with no statistically significant intergroup differences (PFS: HR = 0.99, 95% CI: 0.11–9.18, *P* = 0.993; OS: HR = 0.94, 95% CI: 0.10–9.10, *P* = 0.959). We identified four long-term responders with remarkably prolonged ICI treatment duration >30 months among the patients receiving ICI-based regimens. Detailed individual clinical, molecular and treatment characteristics of these exceptional responders, including age, metastatic sites, PD-L1 TPS status, concurrent gene alterations, prior treatment history, first-line combination regimens and exact ICI treatment duration, are comprehensively summarized in **Supplementary Table 2**. Briefly, all four long-term responders were diagnosed with stage III/IV SMARCA4-deficient NSCLC and received first-line chemoimmunotherapy. No concurrent actionable driver gene mutations were detected in any of these patients, and only one patient had available PD-L1 testing results showing TPS <1%.

The Kaplan-Meier analysis results for PFS and OS across all clinical variables are detailed in **Table 3**. Among all screened clinical factors, elevated CYFRA21–1 was independently correlated with unfavorable PFS and OS (PFS: 11.70 vs NR, Log-rank *P* = 0.017; OS:15.70 vs NR, Log-rank *P* = 0.025). Chemotherapy showed a marginal prognostic effect on OS (Log-rank *P* = 0.050). Other previously analyzed factors including ECOG performance status and immunotherapy receipt also achieved statistical significance in univariate analysis, as described above. No remaining clinical variables were significantly associated with PFS or OS outcomes (all Log-rank *P* > 0.05).

**Table 3:** Survival analysis of PFS and OS across various clinical features and different therapy modality groups.

Factors	mPFS (months) (95% CI)	Log rank *P*	mOS (months) (95% CI)	Log rank *P*
Overall	27.9(11.4-NA)		31.4(11.7-NA)	
Gender, n(%)		0.894		0.736
Female	27.90 (NA - NA)		NA (8.70 - NA)	
Male	15.30 (8.70 - NA)		31.40 (NA - NA))	
Median age at		0.182		0.076
diagnosis (years)				
<60	NA (15.30 - NA)		NA (NA - NA)	
≥60	11.70 (8.30 - NA)		15.70 (8.30 - NA)	
Smoking history, n(%)		0.200		1.00
No	11.40 (4.30 - NA)		6(40.00)	
Yes	NA (11.70 - NA)		9(60.00)	
Performance status group, n(%)		0.014		0.001
0–1	27.90 (15.30 - NA)		NA (31.40 - NA)	
>1	7.80 (1.00 - NA)		7.80 (2.20 - NA)	
Clinical stage		0.696		0.760
I	1.00		2.20 (2.20 - NA)	
II	NA		NA (NA - NA))	
III	NA		NA (4.30 - NA)	
IV	15.30		31.40 (8.70 - NA)	
T		0.457		0.253
1	NA (1.00 - NA)		NA (2.20 - NA)	
2	11.40 (8.70 - NA)		15.70 (8.70 - NA)	
3	NA (15.30 - NA)		NA (NA - NA)	
4	11.70 (4.30 - NA)		11.70 (6.20 - NA)	
N, n(%)		0.118		0.203
0	NA (4.30 - NA)		NA (4.30 - NA)	
1	3.50 (NA - NA)		6.20 (NA - NA))	
2	19.65 (8.30 - NA)		15.70 (8.30 - NA)	
3	NA (11.70 - NA)		NA (11.70 - NA)	
M, n(%)		0.393		0.578
0	NA (4.30 - NA)		NA (4.30 - NA)	
1	15.30 (8.70 - NA)		31.40 (8.70 - NA))	
Pleural Effusion		0.715		0.519
No	NA (6.90 - NA)		NA (6.90 - NA)	
Yes	19.80 (11.40 - NA)		23.55 (11.70 - NA)	
Intralung Metastasis		0.235		0.164
No	27.90 (15.30 - NA)		NA (31.40 - NA))	
Yes	10.20 (6.90 - NA)		10.20 (6.90 - NA)	
Bone metastases		0.848		0.810
No	15.30 (11.40 - NA)		NA (11.70 - NA)	
Yes	27.90 (8.30 - NA)		31.40 (8.30 - NA)	
Brain metastases		0.391		0.210
No	27.90 (8.70 - NA)		31.40 (8.70 - NA)	
Yes	NA (15.30 - NA)		NA (NA - NA)	
Adrenal metastases		0.476		0.590
No	15.30 (11.40 - NA)		31.40 (11.70 - NA))	
Yes	NA (8.70 - NA)		NA (8.70 - NA)	
Pleural metastasis		0.647		0.655
No	27.90 (8.70 - NA)		31.40 (8.70 - NA))	
Yes	NA (11.40 - NA)		NA (15.70 - NA)	
Histological type		0.093		0.056
NSCLC, not otherwise specified	NA (15.30 - NA)		NA (NA - NA)	
Adenocarcinoma	8.70 (4.30 - NA)		8.70 (6.20 - NA)	
Sarcomatoid carcinoma	11.40 (NA - NA)		15.70 (NA - NA)	
PD-L1		0.083		0.261
<1%	27.90 (8.30 - NA)		31.40 (8.30 - NA)	
1%~49%	7.45(2.9 - NA)		10.95(2.9 - NA)	
>49%				
Ki-67		0.978		0.738
≤30%	27.90 (8.30 - NA)		31.40 (8.30 - NA)	
>30%	15.30 (11.40 - NA)		NA (11.70 - NA)	
NSE		0.815		0.514
Normal	27.90 (8.30 - NA)		31.40 (8.30 - NA)	
Elevated	15.30 (11.40 - NA)		NA (15.70 - NA)	
CEA		0.315		0.164
Normal	NA (11.40 - NA)		NA (15.70 - NA)	
Elevated	11.70 (6.90 - NA)		11.70 (6.90 - NA)	
CYFRA21-1		0.017		0.025
Normal	NA (NA - NA)		NA (NA - NA)	
Elevated	11.70 (8.30 - NA)		15.70 (8.30 - NA))	
SCC		0.185		0.131
Normal	27.90 (11.40 - NA)		NA (15.70 - NA)	
Elevated	10.00 (8.30 - NA)		10.00 (8.30 - NA)	
Chest Surgery		0.586		0.433
No	27.90 (11.40 - NA)		31.40 (11.70 - NA)	
Yes	4.30 (3.50 - NA)		6.20 (4.30 - NA)	
Chest Radiotherapy		0.286		0.205
No	11.70 (8.30 - NA)		15.70 (8.30 - NA)	
Yes	27.90 (15.30-NA)		NA (31.40 - NA)	
Chemotherapy		0.092		0.050
No	6.50 (1.00 - NA)		6.50 (2.20 - NA)	
Yes	27.90 (11.70 - NA)		NA (15.70 - NA)	
Immunotherapy		0.106		0.047
No	6.90 (2.90 - NA)		6.90 (2.90 - NA)	
Yes	NA (11.70 - NA)		NA (15.70 - NA)	
Anti-angiogenic targeted therapy		0.837		0.748
No	NA (8.70 - NA)		NA (8.70 - NA)	
Yes	27.90 (8.30 - NA)		31.40 (8.30 - NA))	
Chemotherapy plus immunotherapy		0.146		0.085
No	8.70 (6.90 -NA)		12.20 (6.90 - NA)	
Yes	NA (11.70 - NA)		NA (NA - NA)	
Chemotherapy plus anti-angiogenic therapy		0.778		0.600
No	15.30 (11.40 - NA)		NA (11.70 - NA)	
Yes	27.90 (6.90 - NA)		31.40 (6.90 - NA)	
Chemotherapy plus immunotherapy and anti-angiogenic therapy		0.991		0.943
No	27.90 (8.70 - NA)		31.40 (8.70 - NA)	
Yes	11.40 (8.30 - NA)		15.70 (8.30 - NA)	

We further characterized patients achieving durable clinical benefit with ICI treatment duration exceeding 30 months (n=4). Detailed baseline demographic, molecular, treatment and efficacy information for these long-term responders is summarized in **Supplementary Table 1**. All four patients received first-line chemoimmunotherapy. PD-L1 testing was only available for Case 2, showing TPS <1%, while sufficient tumor tissue for PD-L1 detection was lacking in the remaining three patients. This finding reinforces the paradoxical feature of SMARCA4-deficient NSCLC: durable clinical responses to ICI-based therapy can be achieved irrespective of low or unavailable PD-L1 status.

### 3.4 Safety

Treatment-related adverse events (TRAEs) were graded according to the National Cancer Institute Common Terminology Criteria for Adverse Events (CTCAE). The incidence and distribution of TRAEs in the ICI and non-ICI groups are summarized in **Table 4**.

**Table 4:** Comparison of Adverse Reactions between the ICI and non-ICI cohorts.

Factors	Total (n = 22)	Non-ICI (n = 7)	ICI (n = 15)	*P*
Myelosuppression				0.136
Grade0	8 (36.36)	5 (71.43)	3 (20.00)	
Grade1	7 (31.82)	1 (14.29)	6 (40.00)	
Grade2	3 (13.64)	0 (0.00)	3 (20.00)	
Grade3	3 (13.64)	0 (0.00)	3 (20.00)	
Grade4	2 (9.09)	1 (14.29)	1 (6.67)	
≥Grade3	5(22.72)	1(14.28)	4(26.67)	
Renal dysfunction				0.682
Grade0	19 (86.36)	7 (100.00)	12 (80.00)	
Grade1	1 (4.55)	0 (0.00)	1 (6.67)	
Grade2	2 (9.09)	0 (0.00)	2 (13.33)	
≥Grade3	0(0.00)	0(0.00)	0(0.00)	
Hypothyroidism				1.00
Grade0	20 (90.91)	7 (100.00)	13 (86.67)	
Grade1	1 (4.55)	0 (0.00)	1 (6.67)	
Grade2	1 (4.55)	0 (0.00)	1 (6.67)	
≥Grade3	0(0.00)	0(0.00)	0(0.00)	

Myelosuppression was the most common hematological adverse event across the entire cohort, occurring in 14 of 22 patients (63.64%). Patients in the ICI group exhibited numerically higher rates of any-grade myelosuppression and grade ≥3 severe myelosuppression compared with the non-ICI group (any-grade: 80.00% vs. 28.57%; grade ≥3: 26.67% vs. 14.28%). However, no statistically significant intergroup difference was observed (*P* = 0.136).

Renal dysfunction was limited to mild-to-moderate (Grade 1–2) abnormalities, with no grade ≥3 renal impairment recorded in either group. Immune-related hypothyroidism occurred exclusively in the ICI group, including one Grade 1 case and one Grade 2 case. No significant differences in the incidence of renal dysfunction (*P* = 0.682) or hypothyroidism (*P* = 1.000) were identified between the two treatment arms. Overall, ICI-based regimens demonstrated a safety profile comparable to conventional non-ICI therapy in patients with SMARCA4-dNSCLC.

## 4 Discussion

Accumulating clinical data demonstrate that pathogenic alterations of the tumor suppressor gene*SMARCA4* (including mutations, deletions, and epigenetic modifications) occur in approximately 5%–10% of all NSCLC cases (4). SMARCA4-dNSCLC represents a rare NSCLC subtype characterized by an unfavorable prognosis and distinct molecular, morphological, and immunophenotypic features (5). Consistent with previous epidemiological reports (9), this subtype predominantly affects elderly male smokers, a pattern also observed in the present cohort. Male patients accounted for 95.45% (21/22) of the overall population, with only one female patient enrolled. A total of 15 patients were aged ≥60 years, and more than half of the cohort (59.09%) had a history of tobacco exposure. These consistent demographic characteristics further support the representativeness of our real-world study population.

The metastatic profiles of stage IV patients in our cohort further reflect the aggressive biological behavior of SMARCA4-dNSCLC. The most common metastatic sites included the lung (45.45%), bone (36.36%), brain (22.73%), pleura (13.64%), and adrenal glands (13.64%). Notably, the relatively high incidence of adrenal metastasis observed in our cohort is in line with previous studies, which have verified that SMARCA4-dNSCLC exhibit a significantly higher tendency for adrenal metastasis compared with *SMARCA4*-intact NSCLC (6). Furthermore, our cohort presented high Ki-67 proliferation indices, with 77.27% of patients showing a Ki-67 index > 30%, accompanied by large primary tumor lesions. These clinicopathological features validate the highly aggressive phenotype of SMARCA4-dNSCLC, which is consistent with a previous study by Naito et al., confirming that the loss of SWI/SNF complex subunit expression is correlated with enhanced malignant tumor biological behavior (26).

PD-L1 expression testing was available for only 11 patients in this cohort. Among these patients, seven (63.64%) had negative PD-L1 expression (TPS < 1%), four (36.36%) had low PD-L1 expression (TPS 1%–49%), and no patients exhibited high PD-L1 expression (TPS ≥ 50%). Accumulated evidence has demonstrated that *SMARCA4* alterations in NSCLC are closely associated with PD-L1 downregulation. Schoenfeld et al. previously confirmed a significant correlation between sequencing-identified SMARCA4-mutant and negative or low PD-L1 expression (*P* = 0.03) (5, 18). Notably, despite the unfavorable low or negative PD-L1 profile, SMARCA4-dNSCLC tumors still retain sensitivity to ICIs. As demonstrated in a large pan-solid tumor genomic profiling study covering over 159,000 patients (27), PD-L1 high expression and elevated tumor mutational burden (TMB) represent largely independent biomarkers with weak correlation in NSCLC, including SMARCA4-altered subgroups. Many SMARCA4-deficient tumors present an immune-desert tumor microenvironment characterized by scarce intratumoral CD8^+^ T cells and low baseline PD-L1 expression; however, in parallel, SMARCA4 inactivation as a key SWI/SNF DNA damage response (DDR) gene aberration correlates with significantly higher TMB compared with SMARCA4-wildtype counterparts, generating abundant neoantigens that may still confer ICI responsiveness even under low PD-L1 status. This intrinsic molecular contradiction renders PD-L1 alone insufficient to stratify immunotherapy outcomes for SMARCA4-dNSCLC.

We observed a pattern of rapid recurrence and disease progression in patients with early-stage SMARCA4-dNSCLC after curative resection. Genomic analyses have demonstrated that *SMARCA4*-inactivated tumors exhibit elevated chromosomal instability, which drives aggressive biological phenotypes, early postoperative recurrence, and site-specific distant metastasis (10). In the surgically resected subgroup of our cohort, the overall objective response rate (ORR) and disease control rate (DCR) both reached 50%, whereas the median progression-free survival (mPFS) was only 4.3 months. Notably, one patient with stage I disease developed recurrent metastatic lesions, including adrenal metastasis, merely one month after surgical resection. This clinical case intuitively reflects the dismal prognosis and unique metastatic organotropism of early-stage SMARCA4-dNSCLC, further validating the robust correlation between *SMARCA4* alterations and adrenal metastasis in NSCLC (6). Longitudinal liquid biopsy based on circulating tumor DNA (ctDNA) enables non-invasive dynamic monitoring of minimal residual disease and tumor genomic evolution, and has emerged as a powerful tool for early identification of subclinical recurrence after curative resection. A recent large-scale secondary analysis of the IMpower150 trial demonstrated that rising ctDNA levels of *SMARCA4, STK11* and *KEAP1* prior to radiographic progression predicted inferior durable clinical benefit, highlighting the predictive value of serial plasma molecular profiling for sequencing-identified SMARCA4-mutant NSCLC (28). Considering the retrospective nature of our single-center cohort, serial ctDNA testing was not uniformly performed for enrolled patients, which represents an important limitation of the present study.

Platinum-doublet chemotherapy is uniformly recommended as standard first-line systemic therapy for most patients with advanced driver-negative NSCLC per global clinical guidelines (29), which also represents the conventional backbone regimen for SMARCA4-dNSCLC, though its clinical efficacy remains controversial in this molecular subtype The SWI/SNF complex participates in DNA damage repair, and platinum-based agents exert anti-tumor activity by inducing DNA damage and triggering tumor cell apoptosis (30). Kothandapani et al. reported that stable *BRG1*-knockout cells presented defective DNA double-strand break repair and enhanced sensitivity to cisplatin (31). In contrast, multiple studies have verified that *SMARCA4* deficiency contributes to chemotherapy resistance in NSCLC, as *SMARCA4* loss suppresses chemotherapy-induced tumor cell apoptosis (32). In our cohort, 18 patients received chemotherapy treatment, including two patients treated with pemetrexed monotherapy and the remaining patients receiving platinum-based combination regimens. Despite platinum-doublet regimens being the standard first-line backbone for driver-negative NSCLC, this therapeutic modality carries inherent drawbacks including cumulative myelosuppression, nephrotoxicity, and subtype-specific chemoresistance, alongside its inability to address interpatient genetic heterogeneity (33). A recent large-scale pharmacogenomic landscape study covering over 200,000 Chinese individuals established the Chinese Pharmacogenomic Knowledge Base (CNPKB), and further validated two novel platinum-response genetic markers (AKT2 chr19:40770621 C>G, SLC19A1 chr21:46934171 A>C) via a cohort of 1,019 lung cancer patients (34). This work demonstrates that germline pharmacogene variants significantly alter platinum anti-tumor activity and toxic risk across Chinese patients, indicating that population-tailored pharmacogenomic testing holds great potential to refine individualized platinum chemotherapy. Nevertheless, such high-throughput genomic profiling has not yet been integrated into routine real-world clinical workflows.

In recent years, immunotherapy has achieved remarkable advances in the treatment of lung cancer. Landmark trials including KEYNOTE-024 and KEYNOTE-042 have established immune checkpoint inhibitors (ICIs) as standard first-line therapy for this disease (35, 36). However, its efficacy remains controversial in patients with SMARCA4-dNSCLC. In our cohort, immunotherapy conferred substantial survival advantages. When stratified by treatment, patients receiving ICI-based regimens showed numerically higher ORR and DCR than those treated without immunotherapy (46.7% vs. 14.3% and 60.0% vs. 28.6%, respectively), though the differences were not statistically significant (*P* = 0.193 for both). Kaplan–Meier survival analyses demonstrated a trend toward longer PFS in the ICI group (median PFS: NR vs. 8.7 months, HR = 0.37, 95% CI: 0.11–1.23, *P* = 0.106). Notably, patients treated with ICIs achieved a statistically significant improvement in OS (median OS: NR vs. 8.7 months, HR = 0.28, 95% CI: 0.08–0.98, *P* = 0.047). Similar survival trends were observed in the subgroup of stage IV patients, where ICI-containing therapy was linked to numerically better PFS and OS without significant intergroup differences. Previous literature includes numerous case reports documenting survival benefits from immunotherapy in SMARCA4-dNSCLC patients (37–39). Meanwhile, several retrospective studies have also confirmed that this treatment can prolong OS among such patients (5, 21, 23). SMARCA4-deficient NSCLC harbors intrinsic immune resistance that restricts single-agent ICI efficacy, whereas chemotherapy induces immunogenic cell death to remodel the suppressive tumor microenvironment and synergize with immunotherapy. KEYNOTE-189, KEYNOTE-407 and IMpower series trials validated chemoimmunotherapy as the standard first-line regimen for advanced NSCLC (14, 15, 40), with superior survival outcomes versus monotherapy strategies. Consistent with these pivotal findings, our cohort data demonstrated numerical survival advantages for chemoimmunotherapy, providing real-world evidence to support combination therapy as the preferred first-line strategy for this high-risk molecular subtype.

Our results confirmed that the combination of immunotherapy and chemotherapy achieves superior clinical efficacy for SMARCA4-dNSCLC. The two treatment modalities exert synergistic anti-tumor activity: chemotherapy not only directly kills tumor cells but also reshapes the host immune microenvironment to boost tumor sensitivity to ICIs. Conversely, ICIs reduce intracellular PD-L1 levels and restore tumor responsiveness to platinum-based chemotherapy (41, 42). A representative case in our cohort further validated this synergistic effect: an initially unresectable stage III patient attained pCR after neoadjuvant chemoimmunotherapy. After 12 cycles of postoperative maintenance immunotherapy, the patient maintained long-term disease-free survival for 45.8 months. Consistent with our observations, a recent retrospective study by Peng et al. (in press) evaluated neoadjuvant chemoimmunotherapy in 29 resectable *sequencing-identified SMARCA4-mutant* NSCLC cases, reporting an ORR of 70.4% and a pCR rate of 51.7%. Notably, their work revealed prominent subtype heterogeneity. Histologically, squamous tumors had a far higher pCR rate (83.3%) than adenocarcinomas (28.6%), which were prone to early recurrence. At the molecular level, patients with concurrent *KRAS*/*KEAP1* or *KRAS*/*STK11* mutations formed an immune-cold subgroup with universal relapse. These findings suggest that histological type and concomitant gene mutations are key factors influencing neoadjuvant chemoimmunotherapy outcomes in *SMARCA4*-altered NSCLC, and such heterogeneity should be fully considered in clinical decision-making (43). Concurrent mutations in *KRAS*, *KEAP1* and *STK11* have been proven to correlate with poorer prognosis. Tumors harboring both *KRAS* and *SMARCA4* mutations exhibit reduced CD8^+^ T cell infiltration. Meanwhile, *KEAP1* and *STK11* are recognized as immune-negative driver mutations, which shape an immunosuppressive tumor microenvironment and eventually lead to primary resistance to immunotherapy (9, 12, 41).

Accumulating translational and real-world evidence has verified that concurrent genomic co-alterations including *KRAS, STK11, KEAP1* and *TP53* serve as critical modifiers of tumor biological behavior and immunotherapeutic responsiveness in SMARCA4-deficient NSCLC (44). Concurrent *TP53* loss commonly exacerbates chromatin dysfunction driven by SMARCA4 inactivation, further amplifying immunosuppressive signaling within the tumor microenvironment and attenuating durable anti-tumor responses to ICIs. Meanwhile, KRAS-mutant SMARCA4-deficient tumors exhibit heterogeneous clinical outcomes depending on accompanying co-mutations: *KRAS/STK11* double-mutant cases display markedly inferior survival and minimal ICI benefit, whereas KRAS-mutant tumors without concurrent *STK11* aberration retain partial responsiveness to chemoimmunotherapy regimens (45). Tumor mutational burden (TMB) also acts as an independent predictive biomarker for immunotherapy efficacy in this rare subtype, with higher TMB consistently linked to improved objective response and prolonged disease control after ICI-containing treatment (27). Without *STK11, KEAP1, TP53* and *TMB* data, we cannot further stratify our driver-negative population to dissect how these concurrent genomic events modulate the comparative efficacy of ICI-based versus non-ICI regimens. As a result, we are unable to distinguish whether the observed survival advantage associated with chemoimmunotherapy is modified by high-risk co-mutation subgroups such as *KRAS/STK11* co-alteration, which restricts the depth of biological inference drawn from our real-world outcomes.

Anti-angiogenic agents in combination with immunotherapy have shown preliminary promising results in isolated case reports and small case series (37, 42). Although chemoimmunotherapy combined with anti-angiogenic targeted agents did not yield superior survival benefits compared with chemoimmunotherapy alone in our cohort, a remarkable clinical case deserves attention. One patient with newly diagnosed brain metastases received chemoimmunotherapy plus anti-angiogenic therapy, along with radiotherapy for both primary tumor and intracranial lesions. The patient achieved a partial response (PR) and subsequently received single-agent immunotherapy as maintenance treatment, with a progression-free survival (PFS) of 51.3 months. The patient remained alive and under continuous follow-up at the time of manuscript submission. Mechanistically, anti-angiogenic therapy synergizes with immunotherapy by normalizing disordered tumor vasculature, which alleviates vascular compression and hypoxia within lesions, thereby facilitating the infiltration of cytotoxic immune cells into the immunosuppressive tumor microenvironment (46). Due to limited sample size and insufficient statistical power, our small cohort cannot provide statistically robust evidence of subgroup-specific survival advantages, and further large-scale studies are required to confirm the clinical value of anti-angiogenic therapy for SMARCA4-dNSCLC.

Several limitations of the present study should be acknowledged. First, this was a single-center retrospective analysis with a relatively small sample size of 22 patients, this exploratory retrospective single-center cohort is substantially underpowered owing to the limited sample size. All reported hazard ratios are accompanied by wide confidence intervals, precluding precise quantification of treatment effects. Second, PD-L1 testing was only available in a minority of cases, so we failed to further explore the correlation between PD-L1 expression and therapeutic outcomes. Third, the non-randomized design inevitably introduced selection bias, histological subtype distribution was significantly imbalanced between the ICI and non-ICI treatment groups (*P* = 0.027), adenocarcinoma cases were exclusively enrolled in the ICI-treated group, whereas NSCLC-NOS and sarcomatoid carcinoma cases predominated in patients receiving non-ICI regimens. Although numerically consistent survival trends favoring ICI-based therapy were seen within each histological subgroup, the small sample size prevents robust adjustment for this imbalance. Accordingly, all comparative survival results and hazard ratio estimates reported in this work should be interpreted as hypothesis-generating signals rather than definitive, confirmatory evidence to guide routine clinical decision-making. In addition, we did not systematically analyze the impact of concurrent gene mutations such as *KRAS*, *STK11*, and *KEAP1* on treatment response in this cohort. In recent years, multiple potential synthetic lethal targets for *SMARCA4* have been identified, including *SMARCA2* (47), ataxia telangiectasia and Rad3-related (ATR) (48), and cyclin-dependent kinase 4/6 (CDK4/6) (49). Targeted agents against the SWI/SNF complex, when combined with ICIs or platinum-based chemotherapy, represent a promising therapeutic strategy for this refractory NSCLC subtype. Further studies are warranted to explore molecular subtyping and co-mutation profiles, and large-scale, multicenter prospective clinical trials are urgently needed.

## 5 Conclusion

SMARCA4-dNSCLC is an aggressive and rare NSCLC subtype with unique clinical characteristics. In our cohort, this disease predominantly affected elderly male smokers, accompanied by active tumor proliferation and a high risk of adrenal metastasis. Most cases had low or negative PD-L1 expression, yet patients still derived notable survival benefits from ICI-based therapy. Given the limitations of the small sample size, single-center design, and incomplete molecular analysis, our findings require further verification. Collectively, chemoimmunotherapy is a favorable therapeutic choice for SMARCA4-dNSCLC. Further research focusing on molecular subtyping and synthetic lethal targeted therapies is warranted, and large multicenter prospective studies are needed to establish standardized treatment regimens for this refractory malignancy.

## Data Availability

The raw data supporting the conclusions of this article will be made available by the authors, without undue reservation.

## Supplementary material

Supplementary Table 1 Baseline clinical, molecular and treatment characteristics of long-term responders with SMARCA4-deficient NSCLC receiving immune checkpoint inhibitor-based therapy.
