# Supplementary material for: Immunotherapy plus chemotherapy improves survival in SMARCA4-deficient NSCLC: a real-world cohort study
Source: Front Immunol. 2026 Aug 17;17:1914598. doi: 10.3389/fimmu.2026.1914598 (PMC13526926; doi:10.3389/fimmu.2026.1914598)
Supplement: Supplementary file 1 [file Table1.docx]

Supplementary Figure S1. Kaplan-Meier survival analysis stratified by treatment in patients with stage IV SMARCA4-deficient NSCLC.

(A) Progression-free survival (PFS); (B) Overall survival (OS). Kaplan-Meier curves compare survival outcomes between patients receiving chemoimmunotherapy (Chemo-IO) and those receiving Chemo-IO plus anti-angiogenesis therapy.

(A) The median PFS was 15.3 months in the Chemo-IO group and not reached (NR) in the Chemo-IO plus anti-angiogenesis group (HR = 0.99, 95%CI: 0.11–9.18, P = 0.993).

(B) Median OS was not reached (NR) in both groups (HR = 0.94, 95%CI: 0.10–9.10, P = 0.959). The number of patients at risk at each time point is shown below each curve.
